# Quantitative 3D mapping of the integrated sweat duct-microvasculature unit via fingerprint-guided photoacoustic/ultrasound microscopy

**DOI:** 10.1016/j.pacs.2026.100894

**Published:** 2026-11-02

**Authors:** Zhida Chen, Yi Liu, Dezi Li, Zihao Yang, Wangbiao Li, Meifang Li, Jing Wang, Zhifang Li

**Affiliations:** a Key Laboratory of Optoelectronic Science and Technology for Medicine of Ministry of Education, Fujian Provincial Engineering Technology Research Center of Photoelectric Sensing Application, Fujian Provincial Key Lab of Photonic Technology, Information Photonics Research Center, College of Photonic and Electronic Engineering, Fujian Normal University, Fuzhou, Fujian 350007, China; b Affiliated Hospital of Putian University, Putian, Fujian, China; c PROYA COSMETICS CO., LTD, China; d Key Laboratory of Intelligent Control Technology for Wuling-Mountain Ecological Agriculture in Hunan Province, Huaihua University, Huaihua, Hunan, China

**Keywords:** Photoacoustic microscopy (PAM), Microvasculature, Sweat ducts, U-Net model, Structural similarity index metrix (SSIM), Fingerprint-guided, Sweat duct-microvasculature unit (SDMU)

## Abstract

Cutaneous thermoregulation relies on coordinated interactions between sweat ducts and the dermal microvasculature. However, single-modality imaging cannot simultaneously visualize and characterize this functional coupling. Here, we present a fingerprint-guided dual-modal photoacoustic/ultrasound (PA/US) microscopy framework for three-dimensional mapping of the integrated sweat duct-microvasculature unit (SDMU). This strategy combines high-frequency ultrasound with deep learning (U-Net) to extract sweat duct and fingerprint features; simultaneously, the distribution of microvasculature in PA imaging is accurately localized via structural similarity index metrix (SSIM) analysis of depth-dependent melanin. Crucially, fingerprint-guided non-rigid registration eliminates motion artifacts, enabling dynamic tracking of the identical SDMUs during physiological processes. Quantitative analysis indicated that the functional coupling of microvasculature is highly concentrated within a 75-micrometer radius around sweat ducts, and that the expansion of US-visible sweat ductal structures exhibits a strong positive correlation with microvascular recruitment. Furthermore, a physiology-informed operational dual-response criterion further revealed significant spatial heterogeneity in functional activation, thereby facilitating robust discrimination between activated and unactivated SDMUs. This study not only established the SDMU as a quantifiable functional unit for studying cutaneous thermoregulation, but also highlighted its potential as a candidate biomarker for assessing diabetes-associated neurovascular dysfunction.

## 1 Introduction

Human skin thermoregulation critically depends on the tightly coordinated activity of eccrine sweat ducts and the adjacent cutaneous microvasculature [1]. sweat ducts regulate body temperature through perspiration, which requires an adequate blood supply [2]. Consequently, a rise in body temperature is often accompanied by vasodilation [3]. Furthermore, the morphology of the sweat ducts also changes subtly with variations in temperature [4]. Both sweat ducts and microvessels are functional micro-organs regulated by the sympathetic nervous system, which drives vasodilation and sweat duct activation under thermal stress [5]. Therefore, sweat ducts and microvessels do not operate independently but form a functionally coupled unit coordinated by the sympathetic nervous system. The synergistic mechanism of this “sweat duct-microvascular” functional coupling unit is crucial for maintaining homeostasis in the human body. However, our current understanding of this synergistic mechanism remains insufficient. Synchronous imaging and quantitative characterization of these functionally coupled units are indispensable methods for elucidating the synergistic mechanisms of cutaneous thermoregulation. Current major non-invasive detection and imaging methods focus separately on either sweat ducts or blood vessels. For instance, the ventilated capsule method [6], [7], infrared thermography [8], [9], and optical coherence tomography (OCT) [10], [11] can measure sweat duct function or structure, while OCT angiography (OCTA) [12], [13] and photoacoustic microscopy [14] primarily provide morphological information of blood vessels [15]. The ventilated capsule method has been established as a quantitative way to measure sweating, but it cannot assess the exact function of sweat on the skin and in individual sweat ducts [16]. Mid-wavelength Infrared (MWIR) thermography provides sweat production and sweat pore activation, revealing patterns of sweat circulation [17]. However, the thermal imaging for real-time dynamic monitoring of sweat pores is the limited depth of field of macro lenses, which makes it difficult to clearly visualize the subtle change of sweat pores. OCT enables clear identification and extraction of the spiral structures of epidermal sweat ducts due to their high reflectivity and the significant refractive index contrast with surrounding epidermal tissue. However, OCT is limited by the difficulty of simultaneously capturing high-performance images of both blood vessels and sweat ducts [18]. In addition, OCTA applied inherent motion contrast to differentiate functional vessels from static tissue backgrounds, but vessels in the skin without red blood cell flow cannot be visualized [19] and the image quality of OCTA is highly dependent on the selection of its threshold for blood vessels. Photoacoustic microscopy (PAM) combines laser-induced optical illumination with ultrasound detection to map optical absorption differences in biological tissues [20], which can overcome the issues associated with OCTA in vascular imaging. PAM is non-invasive, achieves high spatial resolution, and provides excellent endogenous contrast through hemoglobin and melanin absorption, thereby allowing detailed visualization of both vascular networks and melanin distribution in vivo [21]. Compared to OCT imaging, it is capable of avoiding severe optical scattering by relying on ultrasonic detection, thereby enabling sensitive detection and visualization of the microvascular structure at the microscopic level [22], [23]. However, PAM cannot provide the structure of the sweat ducts. Consequently, there is an urgent need to develop novel methods capable of achieving simultaneous imaging and quantification of both sweat ducts and blood vessels. To address the aforementioned limitations, this study employed a novel dual-modality PA/US microscopic imaging system to achieve simultaneous mapping and correlation analysis of the “sweat duct-microvasculature unit” at the fingertip, Rather than analyzing these structures in isolation. In this study, the term “ultrasound-derived sweat gland structure” specifically refers to the ultrasound-visible sweat duct/tubular component; the entire anatomical eccrine gland, including its deeper coiled secretory portion, is not directly resolved by the present ultrasound imaging approach. [24], [25].our system provides a comprehensive 3D visualization of the SDMU microenvironment in vivo. To overcome the inherent motion artifacts during physiological monitoring, we implemented a fingerprint-guided non-rigid registration framework, which enables the precise dynamic tracking of identical single SDMU transitioning from a resting state to exercise-induced heat stress. Crucially, utilizing this platform, we quantitatively elucidated the spatial-temporal coupling dynamics of SDMU for the first time. Our analysis reveals that functional microvascular coupling is highly concentrated within a *75* μ*m* core radius centered on the sweat duct. Furthermore, we discovered a pronounced spatial heterogeneity in autonomic regulation: instead of uniform global activation, the sympathetic nervous system employs a highly efficient “selective recruitment” strategy, activating only ∼70% of SDMUs to meet the localized metabolic demands of perspiration. By establishing the SDMU as a functionally coupled unit, this work not only provides new insights into the micro-regulation mechanisms of skin temperature, but also suggests that the SDMU has potential as a functional biomarker for the early detection and assessment of diabetic peripheral neuropathy.

## 2 Methods and materials

### 2.1 Participants and PAM

A commercial dual-modal PA/US microscopy system (WEL5200; Advantest Corporation, Saitama, Japan) was employed in this study [26]. The system is equipped with laser sources operating at wavelengths of 575 nm with a pulse width of 10 ns. The light energy per unit area is less than *2.292 mJ/cm*^2^, which has been confirmed to be significantly below the safety threshold specified in the ANSI Z136.1 standard [27]. The ultrasound transducer operates at a center frequency of 60 MHz, facilitating high-resolution in vivo imaging. The 575 nm wavelength is primarily absorbed by hemoglobin, enabling visualization of the distribution of blood vessels. Co-registered high-frequency ultrasound imaging was performed to provide structural information of fingerprints and sweat ducts. Therefore, this system is capable of simultaneously acquiring vascular and ultrasound images to determine their relative positions.

A total of 10 healthy participants with no history of disease were recruited, all procedures were explained in detail, and written informed consent was obtained from each participant. In addition, during the imaging process, all participants wore protective eyewear to prevent potential eye damage from the laser. The participant’s finger was coated with coupling agent and positioned at the membrane-sealed base of tank filled with water, ensuring consistent and minimal pressure during imaging. The fingertip was irradiated with pulsed light at wavelengths of 575 nm. The scanning step size was set to 15 μm, corresponding to scan areas of 6 × 6 mm^2^, as illustrated in Fig. 1. This research was approved by the Ethics Committee of Fujian Normal University.

**Fig. 1:**
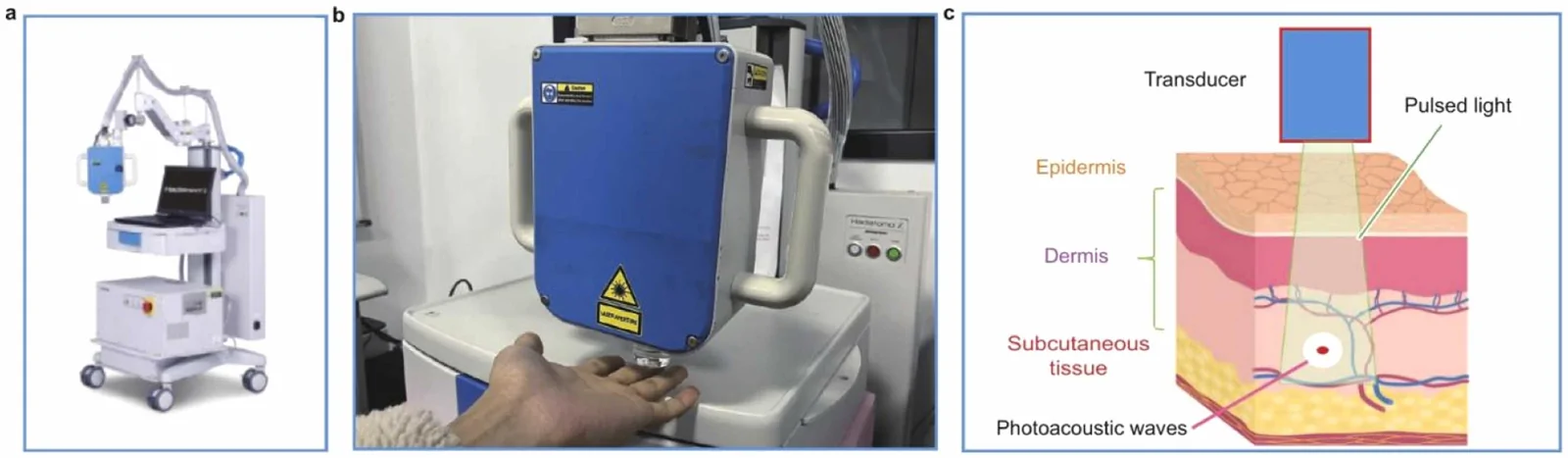
Dual-modal PA/US microscopy for in vivo human fingertip imaging. a,Dual-modal Photoacoustic/Ultrasound microscopy system. b, Imaging of a fingertip skin: a high-frequency ultrasound probe is inserted into a soft immersion cup filled with water, and standard ultrasound gel is used as a coupling medium to ensure contact between the bottom of the cup and the finger for stable acoustic coupling. c, Schematic diagram of the PAM principle for human skin. Pulsed laser light penetrates the skin and is absorbed by hemoglobin, inducing thermoelastic expansion that generates photoacoustic signals detected by the transducer.

### 2.2 Computational workflow for sweat duct-microvessel unit analysis

To accurately characterize and quantify the SDMU, we developed a three-stage workflow for spatial mapping and quantitative analysis (Fig. 2):

**Fig. 2:**
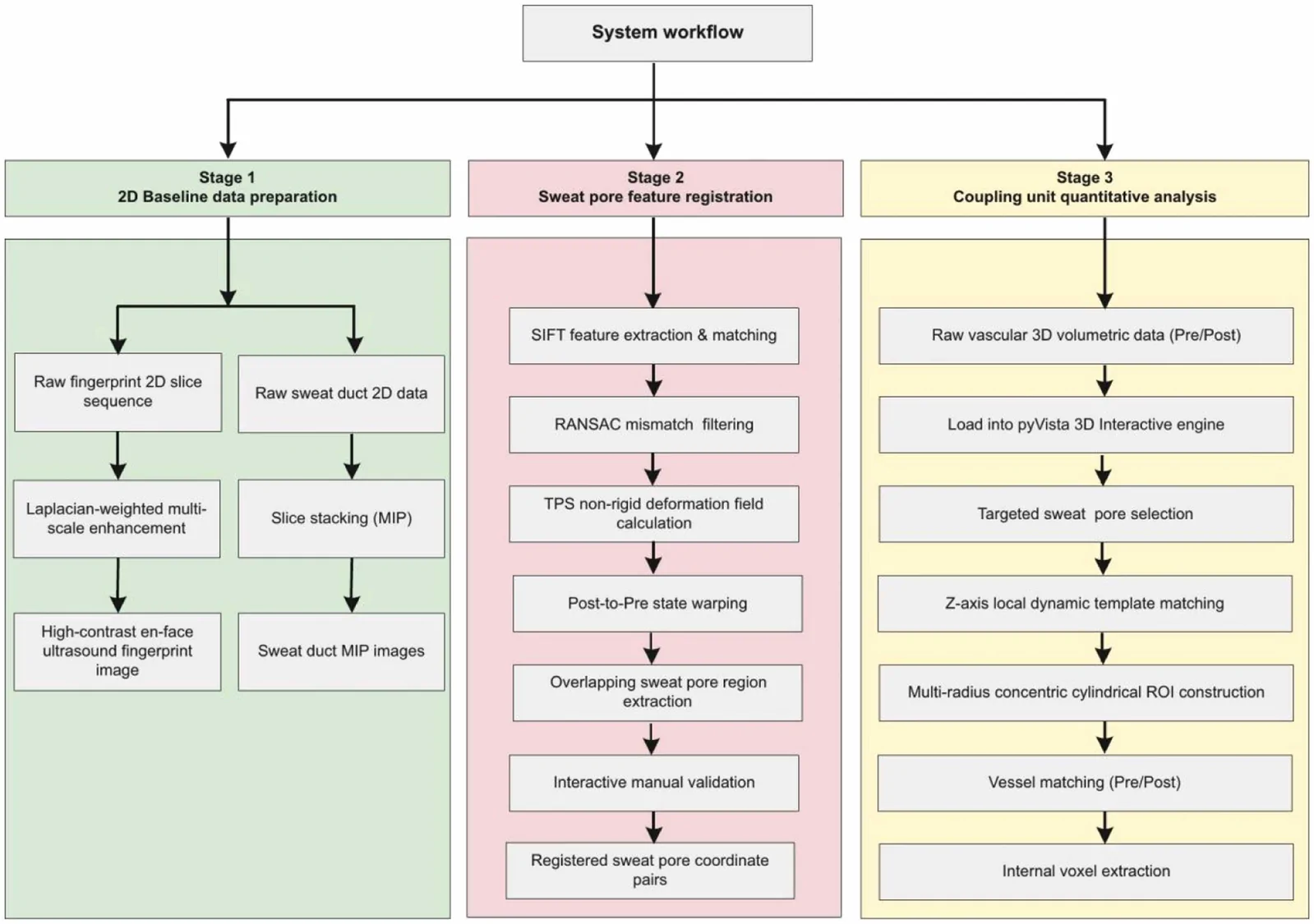
Overview of the computational workflow for spatial mapping and quantitative analysis of SDMUs. The process consists of three main stages: (Stage 1) 2D baseline data preparation, utilizing Laplacian-weighted enhancement and maximum intensity projection (MIP) to extract high-contrast fingerprint and sweat duct features; (Stage 2) Fingerprint-guided non-rigid registration, employing SIFT, RANSAC, and Thin Plate Spline (TPS) transformations to correct motion-induced spatial misalignment between rest and motion states; and (Stage 3) quantitative analysis of coupled units, including PyVista-based 3D visualization, Z-axis dynamic template matching, and concentric cylindrical ROI construction to enable precise extraction of vascular voxels.

1.**Stage 1: Preparation of 2D baseline data.** Initial preprocessing is performed on the raw bimodal data to extract high-fidelity structural landmarks. For the ultrasound image data, a Laplacian-weighted multiscale enhancement algorithm is applied to generate high-contrast en-face fingerprint images. Concurrently, a U-Net model is utilized to obtain cross-sectional sweat duct data and maximum intensity projection (MIP) reconstructions of the en-face morphology of the sweat ducts. These enhanced structural features provide a reliable reference for subsequent spatial registration.

2.**Stage 2: Registration of sweat pore features.** Dual-modal imaging of the fingertip inevitably results in spatial misalignment between the resting (Pre) and post-exercise (Post) physiological states. To address this issue, we developed a non-rigid registration framework guided by ultrasonic fingerprints. First, we applied Scale-Invariant Feature Transform (SIFT) to extract and match keypoints from enhanced fingerprint images, followed by the Random Sample Consensus (RANSAC) algorithm to filter out outliers from failed matches. Next, the Thin Plate Spline (TPS) transformation is applied to compute the non-rigid deformation field, which distorts the post-exercise data to achieve perfect alignment with the pre-exercise state. After interactive manual verification of the overlapping regions, precisely registered sweat pore coordinate pairs are exported.

3.**Stage 3: Quantitative Analysis of the Coupling Units.** Utilizing the registered sweat pore coordinates, the raw 3D volumetric vascular data are spatially mapped and loaded into a PyVista-based 3D interactive interface. Multi-radius concentric cylindrical regions of interest (ROIs) are constructed around each selected sweat duct. A local dynamic Z-axis registration is then applied to compensate for any axial displacement induced by vasodilation. Ultimately, the precisely registered microvascular voxels are extracted to quantitatively evaluate the spatial coupling strength and functional recruitment of the SDMU.

### 2.3 U-Net segmentation of fingertip boundary and sweat ducts

For accurate segmentation of the fingertip boundary and sweat duct structures in ultrasound images, we employed a deep learning network based on the U-Net architecture for dual segmentation tasks. This convolutional neural network (CNN) can precisely locate the boundaries of the curved fingertip in cross-sectional high-frequency ultrasound images, and distinguish sweat ducts from surrounding tissues. This method exhibits outstanding performance, enabling it to serve as an effective solution for adaptive automatic segmentation in medical imaging [28].

This study utilized 2000 manually annotated cross-sectional ultrasound images to segment the sweat ducts and locate the fingertip skin boundary. To enhance the robustness of the model, these ultrasound images were randomly divided into training (70%) and validation (20%) sets. Furthermore, 10% of the images were set aside for rigorous testing. Cross-validation techniques were used to accurately identify performance differences between the training and validation sets, thereby optimizing the hyperparameters. In the experiments, a batch size of eight was configured, and 200 epochs of training were conducted. The RMSprop optimizer was used for model updates, with an initial learning rate of 0.0001 and a decay rate of 0.995. The resulting model achieved a core segmentation metric (Dice coefficient) of ∼98.5% on the independent validation set, which indicates that the segmentation results highly correspond to manual annotations. More detailed architectural specifications and the complete training pipeline can be found in Supplementary Information S1. Fig. 3(a) is an unsegmented ultrasound image containing the coupling film and the finger cross-section, while Figs. 3(b) and 3(c) represent the segmented ultrasound images showing the finger boundary and sweat ducts, respectively.

**Fig. 3:**
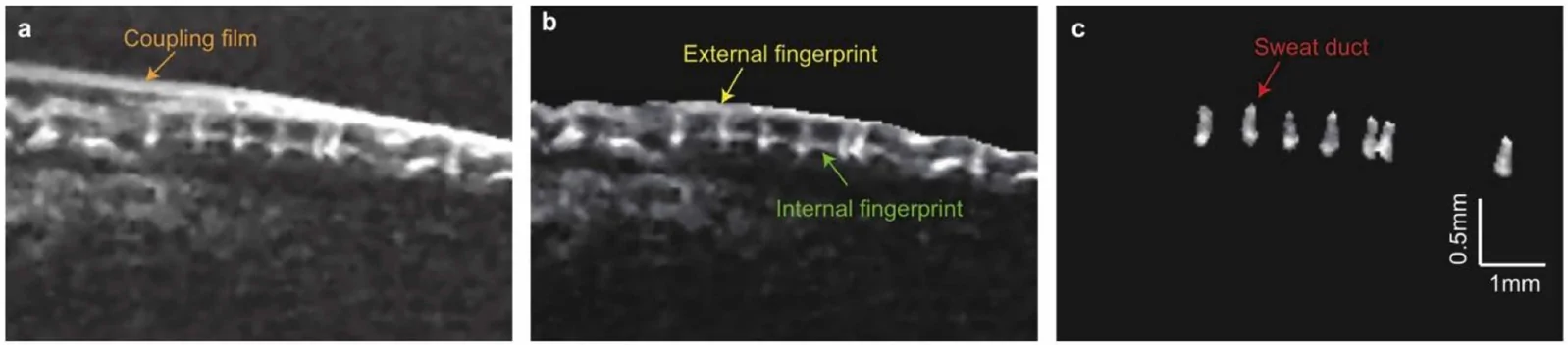
U-Net based segmentation workflow. a, The cross-sectional US image of the fingertip. The orange arrow indicates the coupling film. b, Segmentation results showing the cross-section of the fingertip; the yellow and green arrows represent the epidermal external fingerprint and internal fingerprint, respectively. c, Extracted cross-section of a sweat duct. Scale bar: 0.5 mm (vertical) and 1 mm (horizontal).

### 2.4 Ultrasound image enhancement and sweat duct extraction

The proposed hybrid PA/US technique enables simultaneous visualization of skin structure and microvascular distribution. Within this dual-modality framework, ultrasound signals are primarily used to capture structural information of the fingertip. To optimize the visualization of fingertip boundaries and sweat duct structures in the raw ultrasound data, guided filtering-based image enhancement was applied prior to 3D reconstruction. Guided filtering, originally proposed by He et al. [29], is a highly effective edge-preserving filtering method. Fig. 4 shows an ultrasound image of the detailed structure of the fingertip. Specifically, Fig. 4a displays a three-dimensional reconstructed ultrasound image of fingertip structures, clearly revealing the fingerprint texture on the stratum corneum as well as the detailed morphology of the sweat ducts within the epidermal region.

**Fig. 4:**
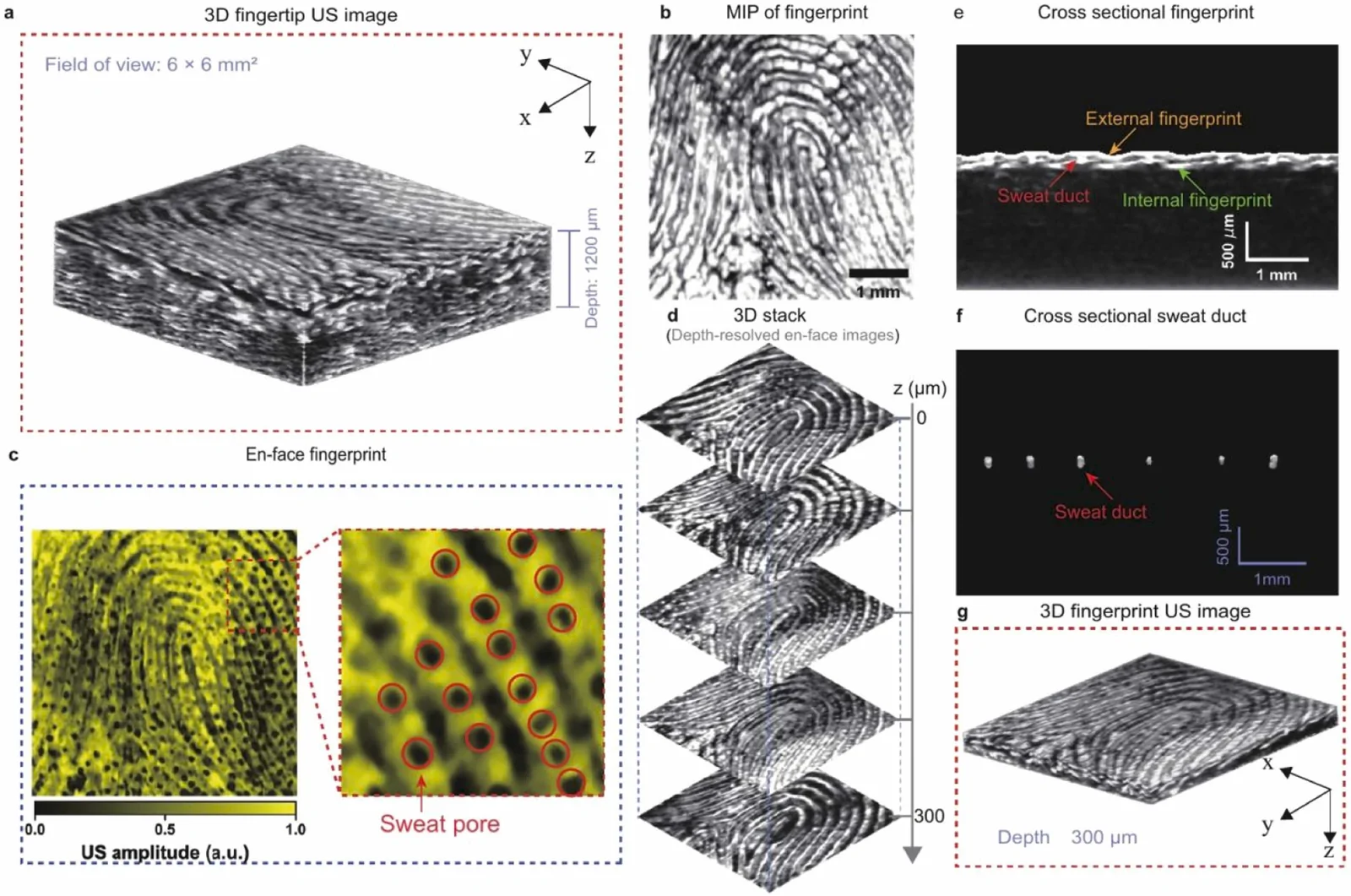
Ultrasound imaging of fingertip structures. a, Three-dimensional volumetric rendering of fingertip structures acquired by high-frequency ultrasound, with an imaging depth of approximately 1.2 mm. b, MIP ultrasound image showing the fingerprint in en-face view. c, En-face ultrasound visualization of fingertip texture and sweat ducts. The enlarged view of the region marked by the red dashed box shows sweat pores (red circles) distributed along fingerprint ridges. d, Depth-resolved en-face ultrasound stack of fingertip skin layers. e, Cross-sectional US image showing the external fingerprint, sweat ducts, and internal fingerprint, indicated by orange, red, and green arrows, respectively. f, Cross-sectional US image of segmented sweat ducts, indicated by the red arrow. g, Three-dimensional rendering of the isolated fingerprint region at a depth of approximately 300 μm. The en-face ultrasound images were acquired over a 6 × 6 mm^2^ field of view with a lateral scanning step size of 15 μm. Cross-sectional ultrasound images were acquired with an axial scanning step size of 12 μm. Scale bars: 1 mm in b; 1 mm (horizontal) and 500 μm (vertical) in e and f, as indicated. The obtained ultrasound images of sweat ducts were further quantitatively analyzed based on the two-sided Mann–Whitney *U* test, as shown in Fig. 10.

Fig. 4b displays the en-face MIP ultrasound image of the fingertip, in which textural information is clearly rendered. Fig. 4c presents en-face ultrasound visualization of fingertip texture and glands. The red dashed box d in Fig. 4c represents an enlarged view of the ultrasonic fingerprint marking area, revealing clearly visible sweat pores (red circles) precisely aligned along the crests of the fingerprint ridges. Fig. 4d illustrates a 3D stack of en face slices, demonstrating the depth-resolved ultrasound imaging of the fingertip skin layers.

Figs. 4e and 4f show the cross-sectional ultrasound images of the human finger and the isolated sweat ducts, respectively. As illustrated in Fig. 4e, the external fingerprint (orange arrows), sweat ducts (red arrows), and internal fingerprint (green arrows) can be clearly distinguished. Fig. 4f further highlights the cross-sectional morphology of the isolated sweat ducts (red arrows). Notably, by combining the volumetric rendering (Fig. 4a) with these precise cross-sectional extractions (Fig. 4f), this represents, to the best of our knowledge, the first demonstration of utilizing high-frequency ultrasound to clearly resolve the in vivo spatial morphology and distribution of sweat ducts within a dual-modal imaging context. Finally, Fig. 4g presents a 3D rendering of the fingerprint region, with a depth of approximately 300 μm. Crucially, the fingerprint ridges and sweat pores serve as structural landmarks, providing essential spatial references for the precise localization and structural matching of the microvascular networks subsequently acquired by the photoacoustic modality.

### 2.5 Vessel localization based on depth-dependent spatial distribution of melanin

To accurately localize the microvascular network within the fingertip, it is essential to first delineate the dermal-epidermal junction (DEJ), as blood vessels are strictly distributed beneath the epidermis. Since melanin is predominantly confined to the epidermal layer, we utilized the Structural Similarity Index Measure (SSIM) [30] to quantitatively evaluate the depth-dependent distribution of melanin across adjacent en-face PA slices acquired at 650 nm. SSIM effectively captures both intensity variations and geometric structural similarities, making it highly robust for tracking the continuity of biological textures along the depth axis. The corrected notation uses sigma-based symbols for the variance and covariance terms in the SSIM calculation.

SSIM was defined as

(1) $$\mathrm{SSIM}(I,I\prime )=\frac{\left(2\mu_{I}\mu_{I\prime }+C_{1})(2\sigma_{\mathrm{II}\prime }+C_{2}\right)}{\left(\mu_{I}^{2}+\mu_{I\prime }^{2}+C_{1})(\sigma_{I}^{2}+\sigma_{I\prime }^{2}+C_{2}\right)}.$$

where *I* and *I′* denote the two compared en-face PA images; *I(n)* and *I′(n)* represent the nth pixel intensity of *I* and *I′,* respectively; *N* is the total number of pixels; *μ*_*I*_ and *μ*_*I′*_ are the mean intensities; *σ*_*I*_^*2*^ and *σ*_*I′*_^*2*^ are the intensity variances; *σ*_*II′*_ is the covariance between *I* and *I′; and* C_1_ and C_2_ are small positive constants used to stabilize the calculation.

$$\mu_{I}=\frac{1}{N}\sum_{n=1}^{N}I(n),$$

$$\mu_{I\prime }=\frac{1}{N}\sum_{n=1}^{N}I\prime (n),$$

$$\sigma_{I}^{2}=\frac{1}{N}\sum_{n=1}^{N}{\left[I(n)-\mu_{I}\right]}^{2},$$

$$\sigma_{I\prime }^{2}=\frac{1}{N}\sum_{n=1}^{N}{\left[I\prime (n)-\mu_{I\prime }\right]}^{2},$$

$$\sigma_{\mathrm{II}\prime }=\frac{1}{N}\sum_{n=1}^{N}\left[I(n)-\mu_{I}\right]\left[I\prime (n)-\mu_{I\prime }\right].$$

Figs. 5a and 5b display the MIP of en-face spatial distributions of melanin at different depth ranges in the fingertip. As shown in Fig. 5a, within the superficial depth of 0–216 μm, melanin exhibits a predominantly structured arrangement along the fingerprint ridges, originating from melanocytes in the basal layer of the epidermis [31]. Conversely, in the deeper region (216–1200 μm, Fig. 5b), the structured melanin signal completely disappears, leaving only background noise. By calculating the SSIM between adjacent depth slices, we obtained the depth-dependent structural stability curve shown in Fig. 5c. In the epidermal region, the continuous melanin structures yield high SSIM values. However, as the depth reaches the basal layer, the melanin signal abruptly terminates, resulting in a drastic decline in structural similarity. This sharp transition point (indicated by the red dashed line at ∼216 μm) serves as a reliable anatomical boundary, accurately demarcating the epidermis from the superficial dermis. Consequently, this melanin-derived DEJ boundary provides a precise depth reference for the subsequent extraction and localization of the underlying dermal vascular map.

**Fig. 5:**
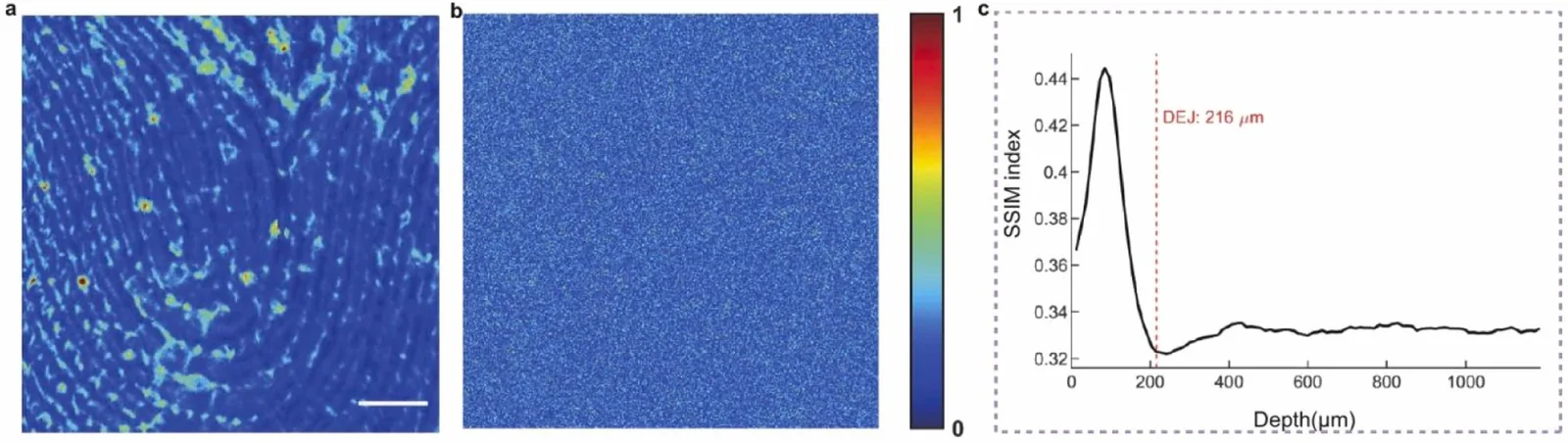
Depth-dependent spatial distribution of melanin for DEJ localization based on 650 nm PA imaging. a, MIP from layers 0–18, b, MIP from 18 to 100 layers, image size = 6 × 6 mm^2^. c, Depth-dependent curve based on structural similarity index. The red dashed line on the left represents the structural similarity curve for regions containing melanin, and the right side represents regions without melanin. This depth-dependent curve can be divided into two regions: the epidermis and the superficial dermis.

### 2.6 Optimized vessel detection workflow for low-contrast imaging

To evaluate and quantitatively analyze fingertip microvasculature, a multi-stage image processing workflow is required to address issues such as low contrast, signal artifacts, and background noise. As shown in Fig. 6, this workflow consists of three main stages: preprocessing, vessel enhancement, and quantitative analysis.

**Fig. 6:**
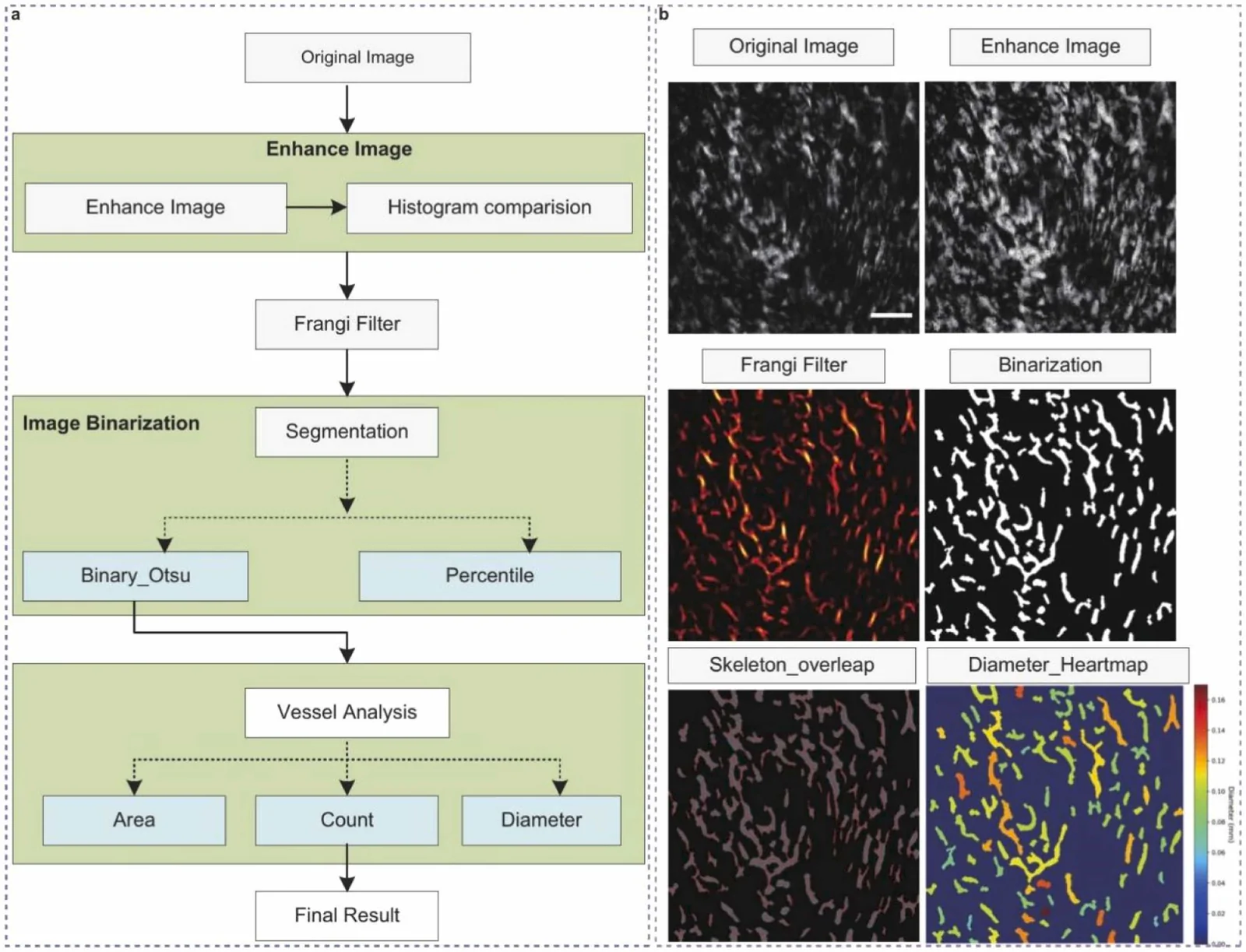
Workflow for photoacoustic microvascular image enhancement and quantitative analysis. a, Algorithm flowchart; b, Representative images from each processing stage. 1) Preprocessing: Preprocessing of the original en-face photoacoustic vascular image using contrast enhancement (e.g., CLAHE) to improve local contrast and make the background signal more uniform. 2) Vascular feature filtering: Apply the Frangi filter to the enhanced image to highlight tubular microvascular structures, generating a vascular feature response map (pseudo-color encoded). 3) Segmentation: Automatic thresholding (Otsu method) is applied to the filtered image to extract a binary vascular mask. 4) Vascular Skeletonization: The segmented vascular network undergoes skeletonization to extract single-pixel topological centerlines (displayed as red lines superimposed on the vascular mask). 5) Vascular Descriptors: Perform geometric and topological analysis based on the skeletonized network. Calculate vessel diameters using the exact distance transformation method and present them as a pseudocolor-coded diameter heatmap. Additionally, extract other parameters (such as area, count derived from statistics of skeletonized pixels) for final quantitative analysis. The complete preprocessing settings, Frangi multi-scale vesselness parameters, post-Frangi thresholding, skeletonization, diameter estimation procedure, and software versions are provided in Supplementary Information S1.

By utilizing raw PA signals acquired at wavelengths of 575 nm (*P*_1_) and 650 nm (*P*_2_), it is possible to distinguish between the absorption of hemoglobin and melanin. Calculating the difference between these two wavelengths yields the primary vascular signal, thereby effectively suppressing interference from melanin in the epidermal texture:

$${\mathrm{PA}}_{\mathrm{vessel}}=P_{1}-P_{2}$$

To robustly highlight the tubular structures characteristic of microvessels, the Frangi multi-scale vessel enhancement filter is applied to the pre-processed images. To obtain clearer distribution of blood vessels at the fingertip and evaluate corresponding quantitative parameters, this paper employs the Frangi filter [32] to enhance features in raw photoacoustic vascular images. Fig. 6a presents our workflow for processing raw vascular images, encompassing image enhancement and the Frangi filter. This algorithm analyzes eigenvalues of the Hessian matrix to construct a multi-scale spatial representation (calculating the vascular response probability at each pixel). The final step involves quantifying the blood vessels based on the binarized results. Fig. 6b illustrates the implementation results. Comparison between the original vascular image and the enhanced histogram reveals more uniform contrast in the enhanced image. The Frangi filter effectively highlights vascular information. By applying skeletonization to the binary mask Fig. 6b, the system generates a topological network representing the vascular midlines Fig. 6b. The diameter heatmap displays the distribution of different vascular diameters, annotated with corresponding colors.

We performed in vivo imaging of the vascular network of the fingertip based on the PA modality of our dual-modal microscopy system. Building upon the melanin-based SSIM boundary detection described previously (Fig. 5), we precisely localized the starting depth of the superficial dermis at approximately 216 μm. Consequently, the vascular imaging focuses on the depth range of 216–1200 μm beneath the skin surface, where the deep vascular network exhibits a dense reticular distribution. Fig. 7a presents the MIP of the vascular network within a 6 × 6 mm^2^ imaging area. To illustrate the depth-dependent distribution, Fig. 7b displays a 3D stack of en-face vascular slices along the Z-axis. Fig. 7c shows a cross-sectional view of the blood vessels in the fingertip, where individual blood vessels are clearly identified (indicated by yellow arrows). It is worth noting that the maximum imaging depth is primarily constrained to 1.2 mm due to the inherent optical attenuation of the PA signal in dense dermal tissue. Finally, Fig. 7d provides a comprehensive 3D volumetric rendering that illustrates the complex spatial distribution of the entire vascular network at the fingertip.

**Fig. 7:**
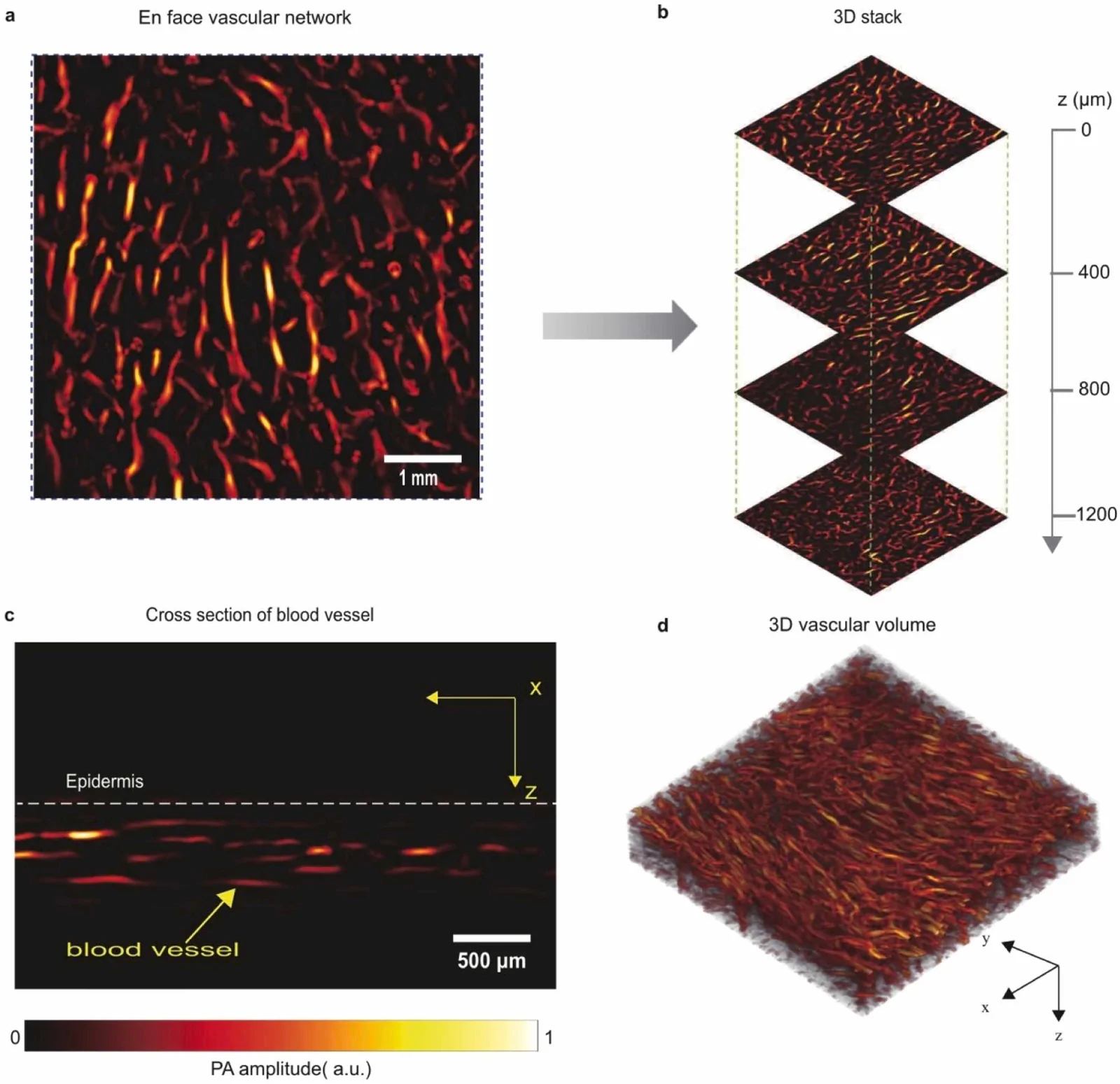
In vivo photoacoustic imaging of the fingertip vascular network. a, En-face MIP of the fingertip microvascular network acquired within a 6 × 6 mm^2^ imaging area. b, Three-dimensional stack of depth-resolved en-face microvascular slices from the dermal vascular imaging range. c, Cross-sectional PA image of blood vessels, with yellow arrows indicating individual vascular structures. d, Three-dimensional volumetric rendering illustrating the spatial distribution of the fingertip vascular network. PA vascular signals were analyzed mainly within the dermal depth range of 216–1200 μm, as determined by SSIM-based DEJ localization. The axial resolution was 12 μm. Scale bars: 1 mm in a and 500 μm in c. The color map represents normalized PA amplitude from 0 to 1 a.u.

### 2.7 Multi-modal image fusion and quantitative evaluation

To fully leverage the complementary strengths of high-frequency ultrasound and photoacoustic imaging, we established a multimodal image fusion framework based on the HSV color space [33]. This framework aims to integrate the epidermal texture and sweat duct morphology information presented in ultrasound images, with the high-contrast distribution of subcutaneous microvasculature in vascular fingerprint images. By employing an HSV color space fusion strategy, this approach enables the simple visual fusion of photoacoustic microvascular images with ultrasound images of sweat duct morphology, clearly illustrating the spatial relationship between sweat ducts and microvessels, thereby facilitating comprehensive observation of the coupled structures. Fig. 8a shows a 3D volumetric rendering of the fingertip after fusion, constructed from 100 superimposed frontal slices. Notably, this 3D visualization clearly demonstrates that the superficial ultrasound fingerprint region (i.e., the epidermis, depth < 216 μm) contains absolutely no vascular signals, whereas a dense microvascular network is strictly confined to the deeper dermal layer. As shown in the fusion workflow (Fig. 8b), this technology enables simultaneous monitoring of epidermal texture, sweat duct morphology, and dermal vascular distribution, thereby providing a comprehensive assessment of both structure and function. Fig. 8c further illustrates the depth-dependent en-face fusion results. Consistent with the 3D observations in Fig. 8a and the anatomical structure of the human fingertip,the fusion results reveal only ultrasonic fingerprint and sweat pore information at a shallow depth of 144 μm, as vascular signals are completely absent at this depth. In contrast, at a depth of 444 μm in the superficial dermis, the dense microvascular network from PA imaging coexists clearly with the deeper ultrasonic structures. Furthermore, Figs. 8d and 8e display cross-sectional fusion images that accurately depict the spatial relationships between the superficial fingerprints, sweat ducts, and underlying blood vessels. Fig. 8f presents a 3D fusion rendering specifically focused on the deep dermal region (depth: 216–1200 μm), where a rich vascular network is distributed beneath the epidermal structures.

**Fig. 8:**
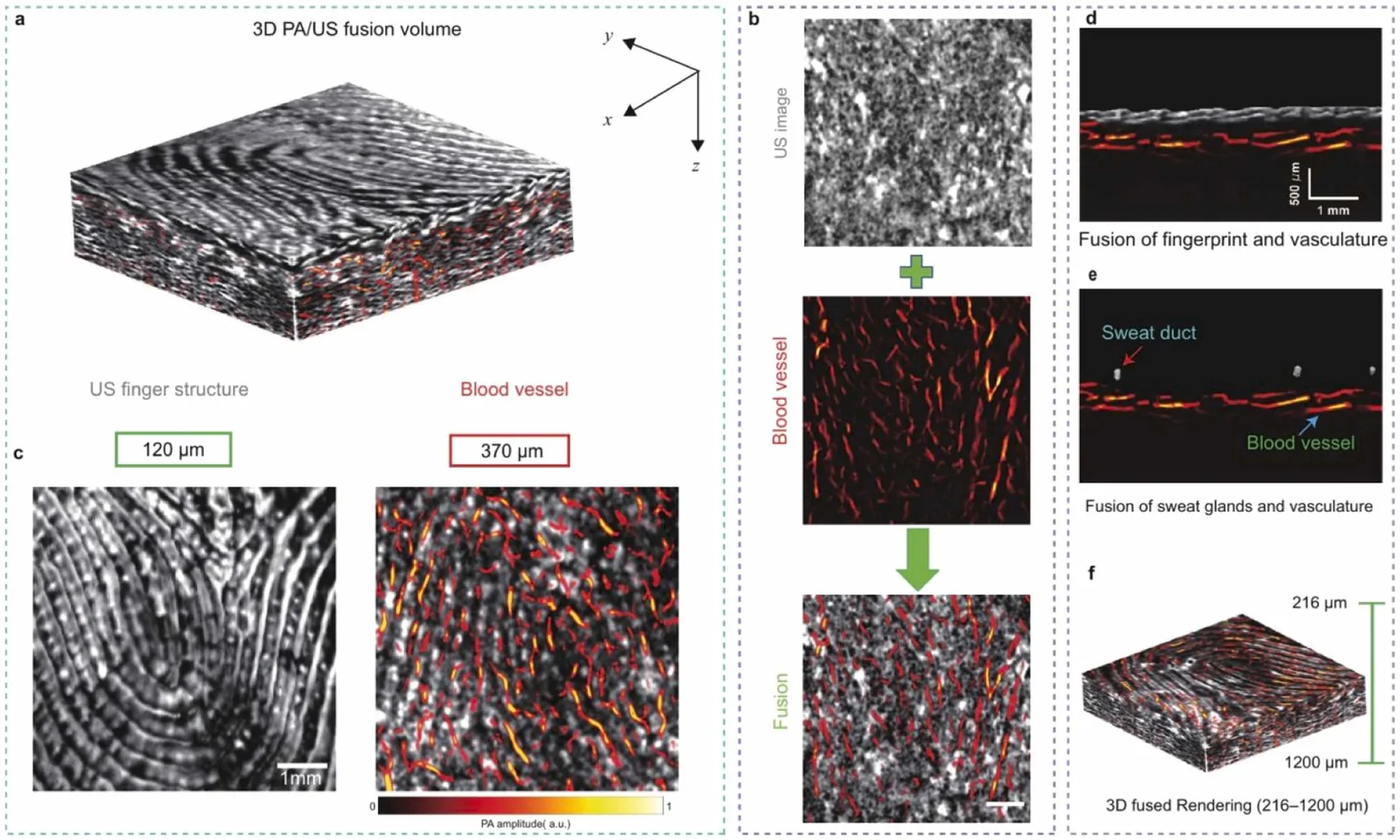
Multimodal PA/US image fusion of the fingertip in vivo. a, Three-dimensional volumetric rendering of the fused fingertip structures(PA vessels in red/yellow, US structures in grayscale), clearly showing the absence of blood vessels in the superficial ultrasound fingerprint region. b, An HSV-based multimodal en-face fusion workflow that integrates ultrasound structures with the corresponding PA vascular network. c, Depth-resolved multimodal fusion image. At a depth of 144 μm (epidermis), only ultrasound fingerprint information is present; at a depth of 444 μm (dermis), the PA vascular network coexists with the ultrasound structures. Scale bar: 1 mm. d, Cross-sectional fusion image showing the spatial relationship between the fingerprint layer and the underlying vasculature. e, Cross-sectional fusion image highlighting the distinct ultrasonic sweat ducts and PA vasculature. f, 3D fusion rendering specifically isolating the depth range from 216 to 1200 μm, demonstrating the deep vascular network beneath the skin surface.

### 2.8 Ultrasound fingerprint-guided non-rigid registration and validation for sweat duct dynamic tracking

To eliminate positioning errors caused by nonlinear deformation of fingertip tissue during thermal stress challenges. As shown in Fig. 9a, the Scale-Invariant Feature Transform (SIFT) [34] combined with Random Sample Consensus (RANSAC) [35] spatial constraints effectively eliminates false matches caused by repetitive fingerprint textures, ensuring the extraction of highly authentic anatomical anchor points.

**Fig. 9:**
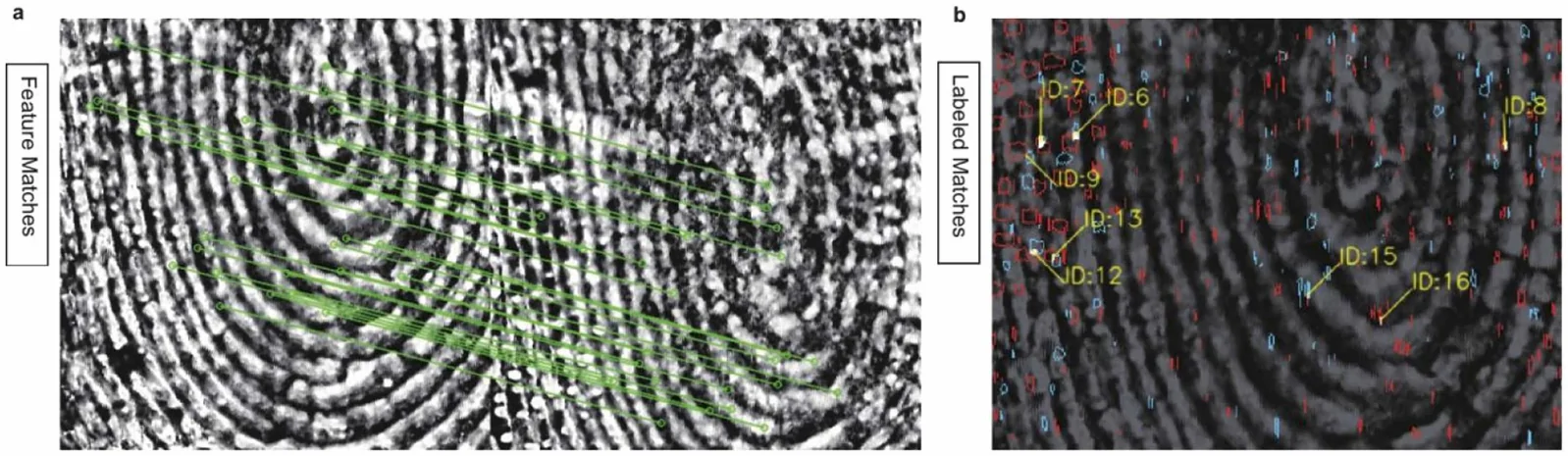
Visual validation of fingerprint-guided non-rigid registration and dynamic sweat duct tracking. a, Feature Matches: RANSAC-filtered SIFT keypoint pairs (green lines) demonstrating highly accurate anchor point detection across resting and post-exercise fingerprint en-face images. b, Labeled Matches: Pseudo-color overlay confirming the successful spatial co-localization and continuous tracking of individual sweat duct units (e.g., ID8 and ID15). The registered fingerprint serves as a rigid structural reference, accurately aligning the pre-exercise (cyan) and post-exercise (red) sweat duct masks.

Before registration, the mean Euclidean distance (MED) between feature points was approximately 18.25 pixels. After performing thin-plate splines (TPS) [36] non-rigid registration, the error significantly decreased to 1*.*38 ± 0*.*3 pixels. This demonstrates the algorithm’s pixel-level accuracy in eliminating motion-induced spatial distortion. Ultimately, this structural registration enables the dynamic tracking of sweat ducts. In the labeled pseudo-color image (Fig. 9b), the matched fingerprint regions serve as structural references, accurately aligning the pre-exercise (cyan) and post-exercise (red) sweat duct masks. The corresponding annotations highlight specific targets (e.g., ID:8 and ID:15), validating the algorithm’s robust capability to continuously and accurately track individual sweat duct units during dynamic physiological processes.

## 3 Results

### 3.1 Exercise-induced sweat duct and microvascular changes

We first assessed changes in the morphology of sweat ducts and blood vessel distribution under different physiological conditions. Fig. 10 shows the detailed quantitative results of sweat duct morphology before and after exercise.

**Fig. 10:**
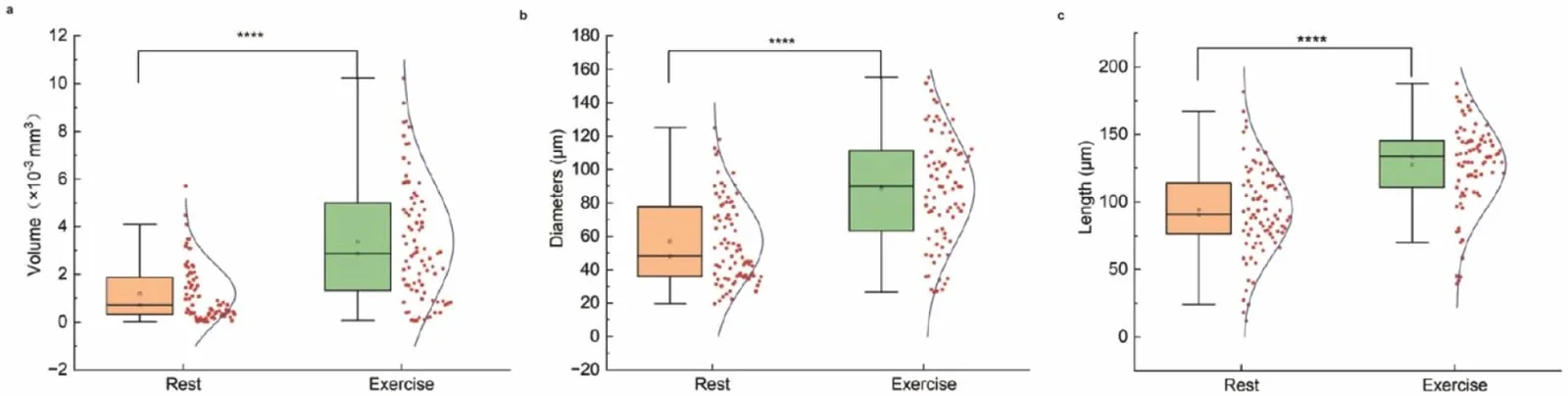
Box plots with normal distribution curves of US-visible sweat ductal structure metrics under rest and exercise conditions. a–c, Variations in reconstructed volume (a), equivalent diameter (b), and length (c) of segmented US-visible ductal/tubular structures with statistical significance (**** *P* < 0.0001) based on the two-sided Mann–Whitney *U* test.

Following exercise, due to active sweat secretion, the segmented sweat duct exhibited significant morphological changes. As shown in Fig. 10a–c, the volume, diameter, and length of these structures increased significantly (p < 0.0001), suggesting exercise-associated morphological responses of the US-visible ductal component, potentially associated with transient luminal volume changes and sweat transport through the eccrine ductal pathway during active sweating [37]. It should be noted that the ultrasound segmentation in this study primarily captured US-visible sweat ductal structures rather than the entire anatomical eccrine sweat duct, particularly the deeper coiled secretory portion.

Our quantitative vascular analysis revealed that overall vascular density significantly increased during exercise compared with rest (*P* < 0*.*001, Fig. 11a). In contrast, the mean vessel diameter and total vessel count did not show statistically significant changes (ns, Fig. 11b,c). These findings indicate that exercise-induced fingertip microvascular responses are mainly reflected by increased PA-detectable representation of perfused microvascular structures rather than uniform enlargement of individual vessels. Although the overall quantitative results confirm that sweat ducts and their surrounding microvasculature are activated during heat stress, evaluating only the overall averages does not allow us to determine whether each local sweat ductal structure is spatially coupled with a matched microvascular response. Therefore, we further evaluated individual sweat duct and their associated vascular regions as paired SDMUs.

**Fig. 11:**
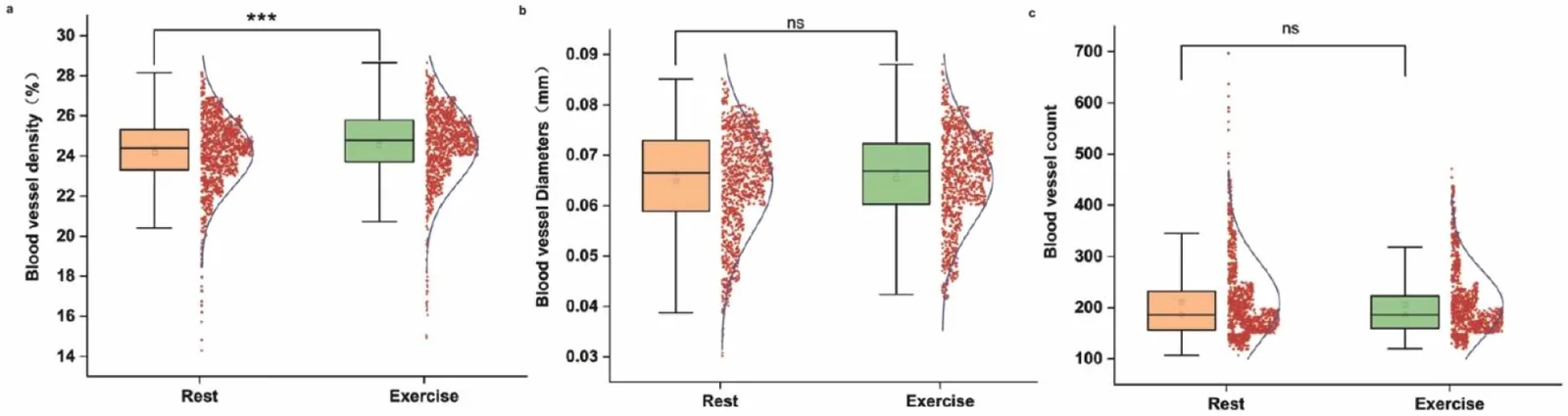
Quantitative statistical comparison of fingertip microvascular parameters between resting and exercise states. a–c, Statistical comparison of vascular density (a), vascular diameter (b), and vascular count (c) between the resting state (orange) and the exercise state (green). Statistical significance was determined using the Mann–Whitney *U* test (*** *P* < 0*.*001; ns, not significant).

### 3.2 Spatial and functional characterization of the SDMU

To investigate the coupling mechanisms between individual sweat ducts and underlying microvasculature, we must first ensure that these dynamic structures are absolutely spatially aligned across different physiological states. Following global registration guided by ultrasound fingerprints and sweat pores, we introduced a locally guided photoacoustic vascular template matching algorithm (Fig. 12). Using the binarized topological features of microvessels at rest as a reference template (Fig. 12a), we identified the matched vascular regions after movement, as shown in Fig. 12d. These two registration steps effectively eliminated the effects of finger tremors and post-motion vasodilation, ensuring that our quantitative analysis was strictly confined to identical SDMUs. Fig. 12e illustrates the 3D coupling unit structure, where we can observe the three-dimensional morphology of the sweat ducts, the associated vascular distribution, and photoacoustic intensity information. After matching vascular units before and after exercise, we sought to define the optimal spatial boundaries of this functional coupling. We quantified coupling intensity based on integrated intensity (defined as vascular density × average signal intensity) and calculated the motion-induced differences (Intensity), which reflect the degree of vascular response. As shown in Figs. 13a and 13c, we evaluated the difference in coupling intensity at multiple radial distances (R2 to R15) from the vascular region beneath the sweat ducts. Analysis indicates that the relationship between the coupling intensity difference and radius does not follow a linear decay; instead, the most significant and consistent microvascular recruitment peaks near the R5 radius (approximately 75 μm). Furthermore, an evaluation of the coupling depth for different sweat ducts at this optimal R5 radius (Figs. 13b and 13d) shows that the strongest vascular response occurs at a physical depth of 1100 μm.

**Fig. 12:**
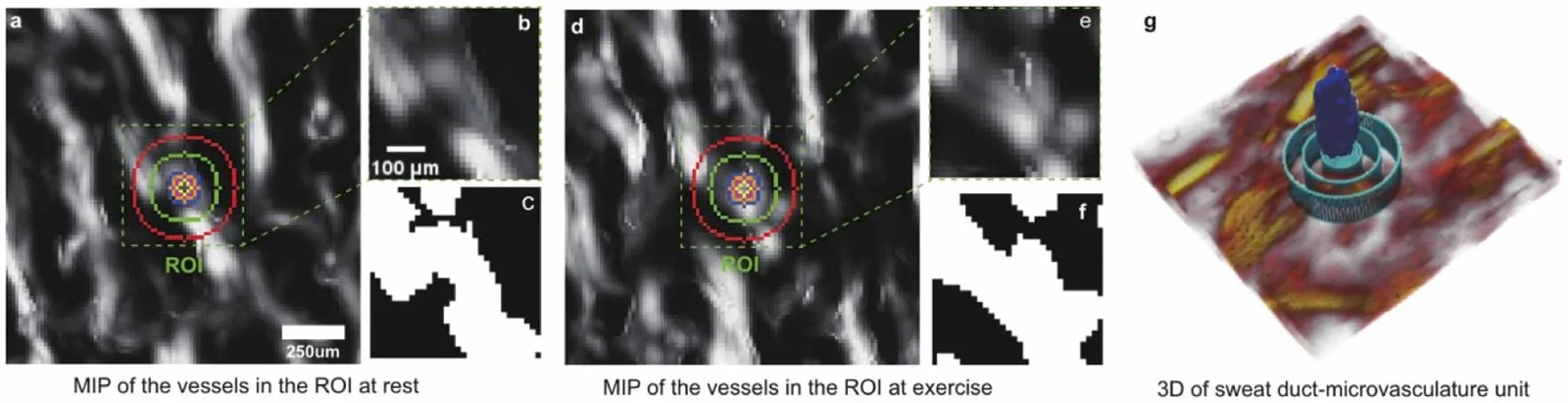
Local template matching and 3D visualization of the SDMU. a, d, MIP of the microvascular network at rest (a) and after exercise (d). Concentric rings indicate the multiscale ROIs centered on the sweat duct. b, e, Magnified views of the green dashed bounding boxes in a and d, respectively. representing the local search areas for template matching. c, f, Corresponding binarized topological features used for the spatial alignment between the two physiological states. g, 3D volumetric rendering of the aligned SDMU, displaying the segmented sweat duct and its associated microvasculature.

**Fig. 13:**
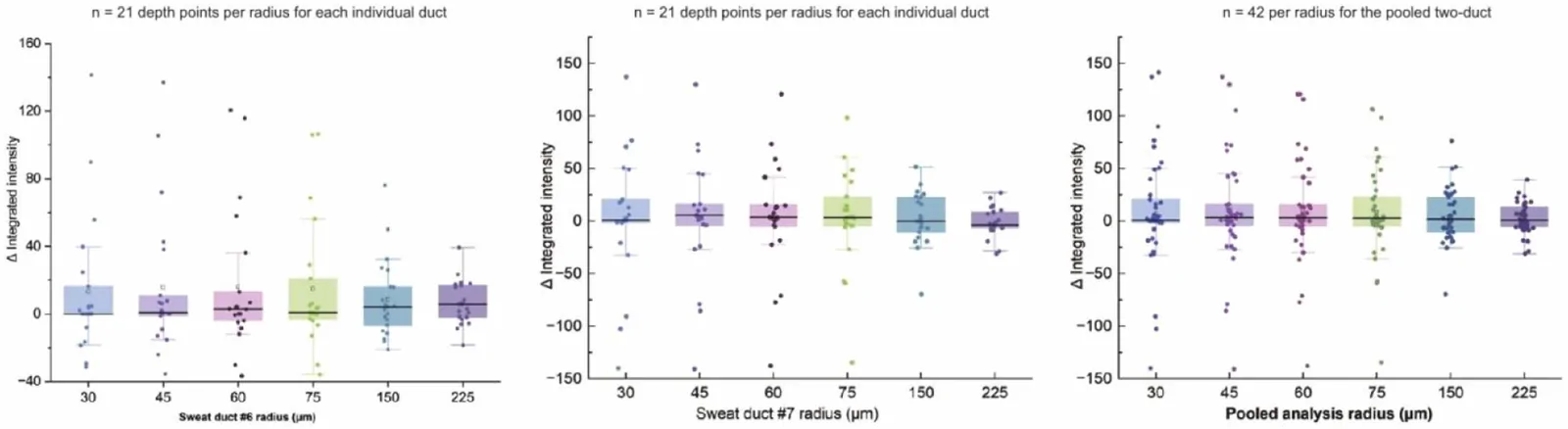
Boxplots illustrating the Integrated intensity differences (Intensity, Exercise minus Rest) of the underlying microvasculature at the base of the sweat duct across varying radial boundaries (30–225 μm). Data are shown for (a) a representative single sweat duct (duct #6), (b) another individual sweat duct (duct #7), and (c) Pooled statistical analysis of data from both sweat ducts (#6 and #7).

As shown in Fig. 13 and Fig. 14, we identified the optimal spatial boundary for microvascular coupling, where the coupling strength peaks at a radial distance of R5, as well as the optimal coupling depth for the vessels within this region. To explicitly validate the reliability of the selected boundaries and demonstrate that US-visible ductal structures and the microvessels beneath them can be robustly analyzed as an SDMU, we performed quantitative correlation analyses within this optimized spatial window between the diameter and volume of the US-visible ductal structures and the integrated intensity of the associated vessels.

**Fig. 14:**
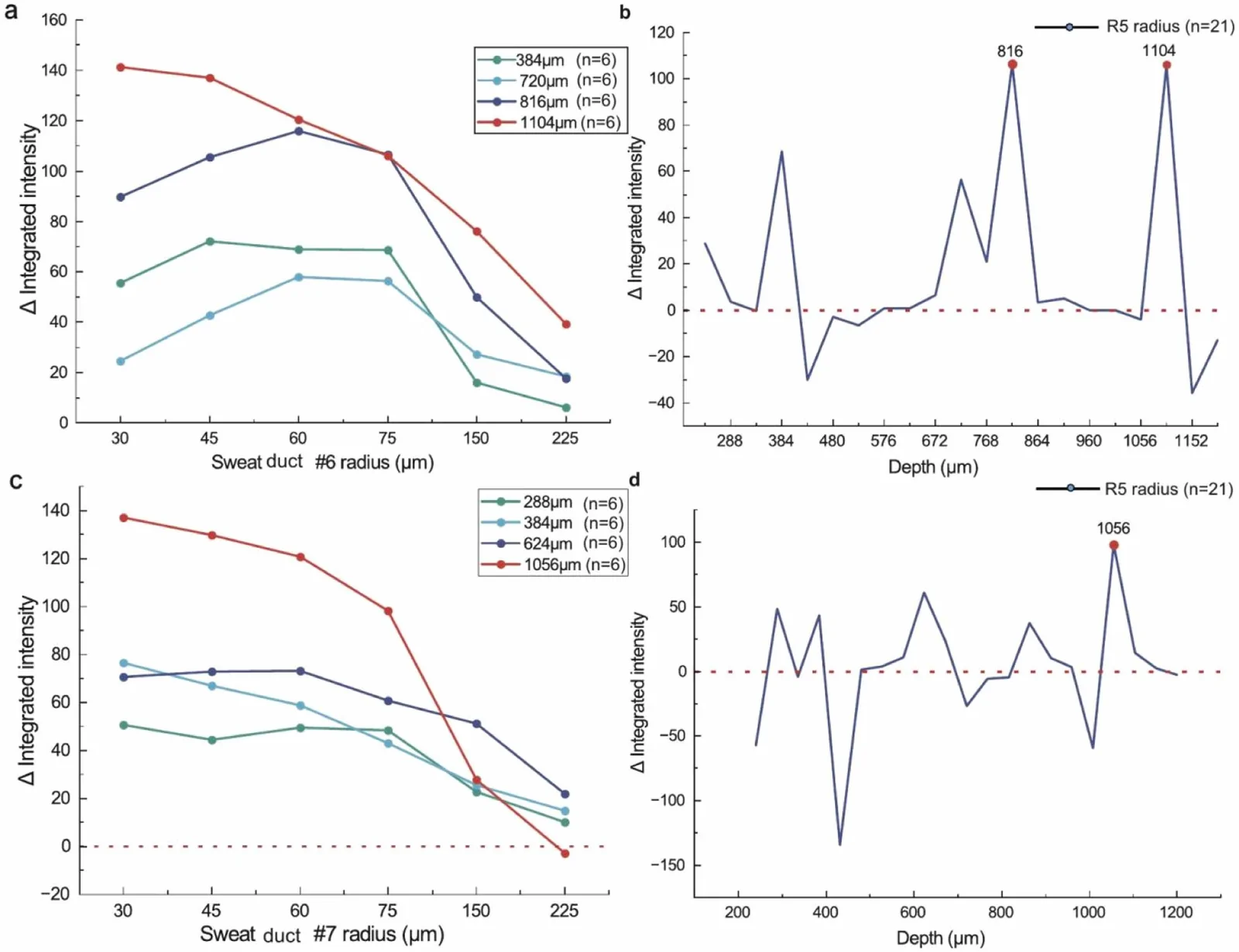
Multiscale and depth-resolved optimization of microvascular coupling intensity. To determine the optimal spatial boundaries of the SDMU, we analyzed variations in coupling intensity for randomly selected individual sweat ducts at different radii and depths. a, Depth-resolved radial analysis for sweat duct #6. Four representative axial depths (384, 720, 816, and 1104 μm) are shown, with six radial-boundary points per depth curve (30, 45, 60, 75, 150, and 225 μm; n = 6 points per curve). b, Depth-resolved profile of coupling intensity at the optimized R5 radius for sweat duct #6 (n = 21 axial depth points). c, Depth-resolved radial analysis for sweat duct #7. Four representative axial depths (288, 384, 624, and 1056 μm) are shown, with six radial-boundary points per depth curve (n = 6 points per curve). d, Depth-resolved profile of coupling intensity at the optimized R5 radius for sweat duct #7 (n = 21 axial depth points). Each marker represents one depth-specific or radius-specific integrated-intensity difference from the paired rest/exercise dataset, rather than a mean across independent biological replicates; therefore, SD or SE error bars are not applicable to individual markers in this representative depth-resolved analysis. The optimal coupling response consistently peaks or remains significant at a radius of R5. The microvascular functional response peaks at an axial depth of approximately 1100 μm, representing the zone of maximal spatial-functional coupling. This depth is close to the expected deep dermal region where eccrine secretory coils are located.

As shown in Fig. 15, a robust positive correlation was observed between the morphological changes of US-visible ductal/tubular structures and the corresponding microvascular response within the optimized R5 coupling region. The normalized change in maximum diameter (Fig. 15a, *r* = 0*.*660*, p* = 0*.*00047) and average diameter (Fig. 15b, *r* = 0*.*621*, p* = 0*.*0012) both showed significant positive correlations with functional microvascular recruitment. Most notably, the three-dimensional volume change of US-visible ductal structures (Fig. 15c) showed the strongest correlation (*r* = 0*.*701*, p* = 0*.*00013). This stepwise increase in correlation strength indicates that three-dimensional volume provides a comprehensive US-derived descriptor of ductal/tubular morphological changes within the optimized SDMU window. Most importantly, the significant correlation observed within the R5 radius supports the reliability of the selected spatial boundary. These findings indicate that post-exercise ductal morphological changes and local vascular recruitment are spatially associated within the optimized SDMU, supporting the rationale for treating these co-registered structures as an imaging-defined functional unit.

**Fig. 15:**
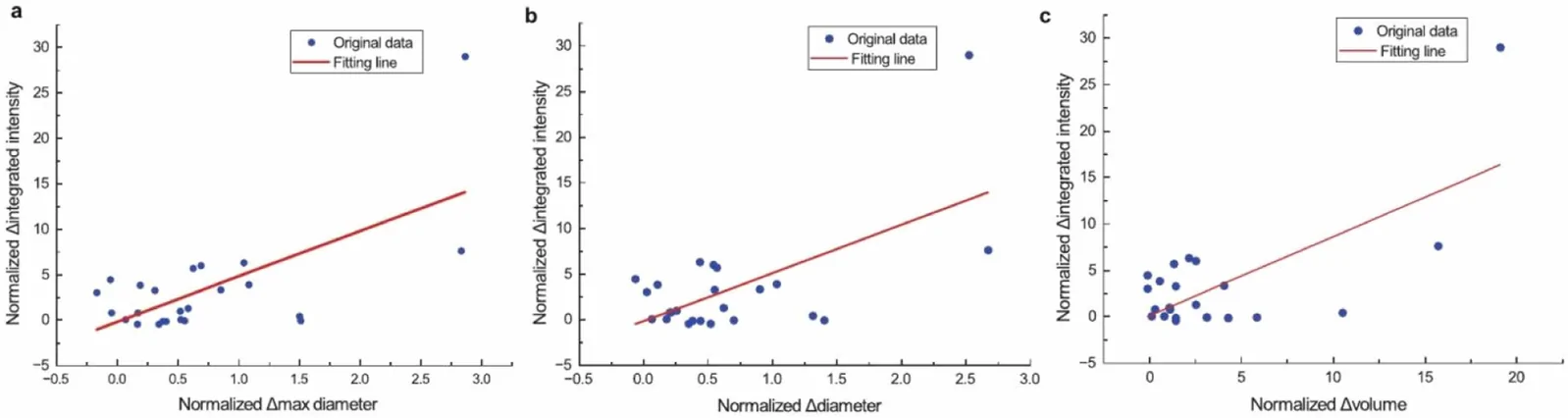
Quantitative correlation analysis validates the SDMU within the optimized spatial boundaries. Pearson correlations were calculated across n = 24 paired SDMUs with complete matched US morphometric and PA microvascular measurements for maximum diameter, average diameter, and total volume. *T*he scatter plots illustrate Pearson correlations between normalized morphological changes in sweat duct and the functional response of the underlying microvessels; the data were extracted from the optimized coupling radius (75 μm) and depth. The correlation analysis included three morphological metrics: (a) maximum diameter (*r* = 0.660, *p* = 0.00047), (b) average diameter (*r* = 0.621, *p* = 0.0012), and (c) total volume (*r* = 0.701, *p* = 0.00013). The significant correlations support the reliability of the R5 coupling boundary and the analysis of US-visible ductal/tubular structures and associated vessels as a co-registered SDMU. The red solid line indicates the ordinary least-squares (OLS) linear regression fit. Normalized differences [calculated as (exercise – rest) / rest] were applied to reduce baseline anatomical heterogeneity.

### 3.3 Spatial heterogeneity and selective activation of SDMUs

The rationale of this classification was derived from established physiological principles of human thermoregulation. During thermal stress or exercise, eccrine sweat secretion is primarily mediated by sympathetic cholinergic activation, representing one of the major effector mechanisms for heat dissipation. In parallel, cutaneous blood flow is regulated through thermoregulatory vasomotor responses, including sympathetic control of active vasodilation, which increases local skin perfusion to facilitate heat loss [38]. Therefore, sweating and cutaneous vascular recruitment represent two coordinated but distinct physiological outputs of the thermoregulatory system. Moreover, sweating responses are not solely determined by the secretory capacity of individual glands, but also depend on the recruitment of functionally active sweat glands across the skin surface, resulting in substantial spatial heterogeneity in glandular activation during thermal challenges [39].

Based on this physiological coupling between eccrine gland activation and cutaneous vascular regulation, we defined an SDMU as exhibiting a coordinated activation response only when both imaging-derived criteria were simultaneously satisfied. First, the normalized change in ultrasound-visible sweat duct morphology was greater than zero (Δ > 0), indicating local structural expansion associated with increased sweat duct activity. Second, the change in PA-derived microvascular coupling intensity within the optimized 75 μm radial boundary was greater than zero (Δ > 0), indicating enhanced local vascular recruitment. SDMUs that failed to meet either criterion were classified as not satisfying the dual-response criterion. This operational definition therefore does not assume that all sweat ducts are uniformly activated, but instead provides an imaging-based framework to identify spatially localized duct–vascular units exhibiting coordinated functional responses during thermal stress.

Based on this strict definition, we conducted a longitudinal analysis of 29 paired SDMUs on a single fingertip. We observed significant spatial heterogeneity in the activation process: approximately 70% (20/29) of SDMUs were coordinated activation activated, while the remaining 30% (9/29) remained dormant. Activated units exhibited synchronous vasodilation and robust local microvascular perfusion, whereas non-activated SDMUs showed almost no morphological or vascular responses.

Representative 3D volumetric visualizations of these distinct phenotypes are provided in Fig. 16. Activated SDMUs (Fig. 16a1–a4) are characterized by enlarged sweat ducts (blue) and dense, high-intensity microvascular networks (red-yellow) concentrated around the sweat duct. In contrast, the non-activated SDMUs shows no significant changes in sweat duct volume, vascular density, or photoacoustic intensity, as shown in Fig. 16b1 and 16b3. These results confirm that during heat stress, SDMUs are not uniformly activated by the sympathetic nervous system. Instead, it employs an efficient strategy of selective recruitment, activating only a spatially specific subset of SDMUs to meet local thermoregulatory demands while conserving systemic blood volume and metabolic energy. This heterogeneous regulatory mechanism is fundamental to maintaining cutaneous thermal homeostasis. These findings are consistent with research indicating that not all eccrine sweat ducts serve functional roles.

**Fig. 16:**
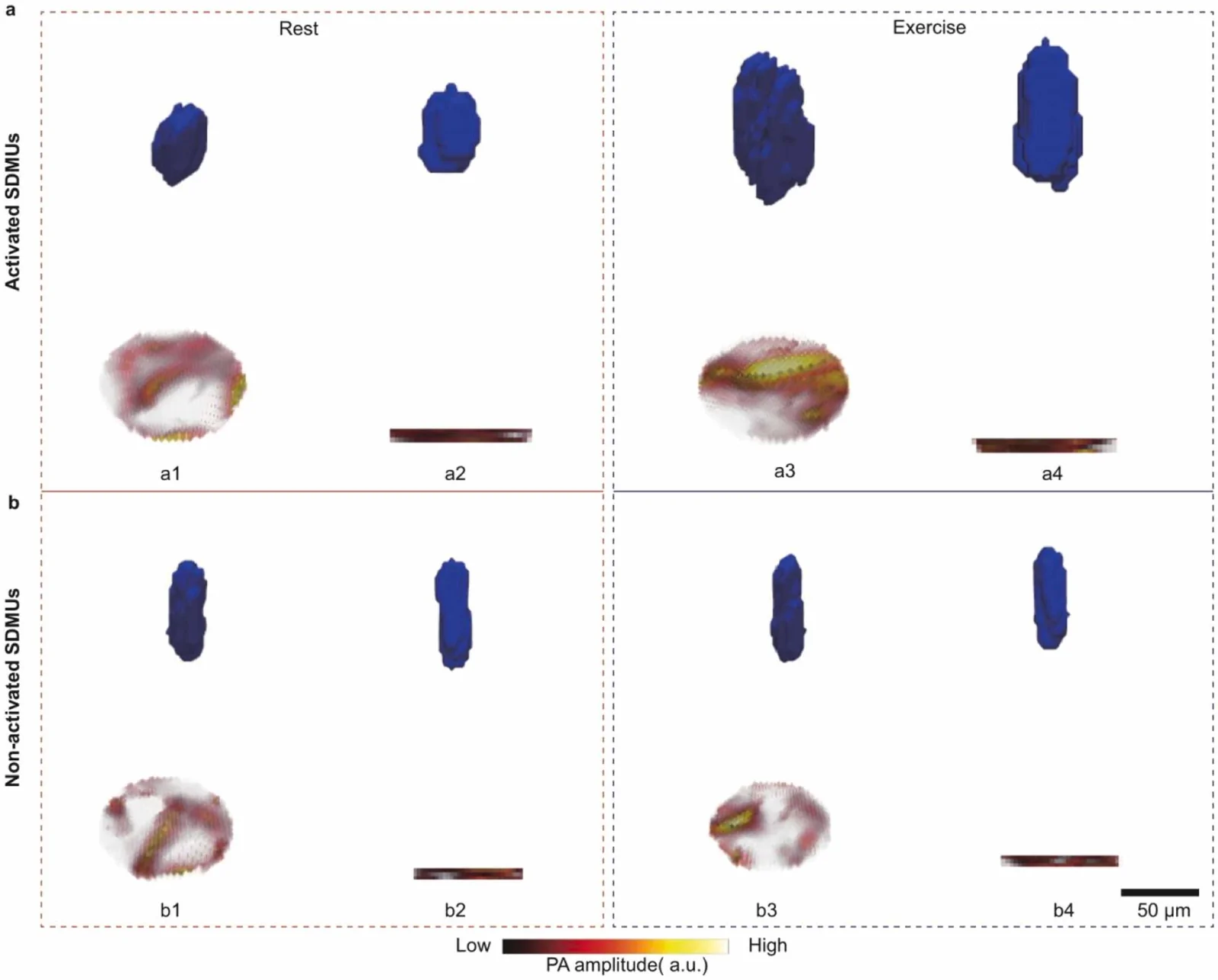
Representative 3D volumetric visualizations of SDMU functional heterogeneity under in vivo heat stress conditions. (a1–a4) Typical examples of SDMUs functionally activated following heat stress. Activated units are characterized by significant sweat duct dilation (blue), and within the local vascular coupling volume, extensive recruitment of surrounding microvessels is observed, accompanied by increased PA signal amplitude (red–yellow). (a1, a3) show paired frontal views of the same set of SDMUs before and after exercise; (a2, a4) show left-side views of the SDMUs. (b1–b4) show representative dormant (inactive) SDMUs, in which the sweat duct morphology is stable and local peri-ductal microvascular perfusion is markedly sparse compared to the corresponding structures in the activated state. The blue segmented structures represent sweat ducts reconstructed via high-frequency ultrasound (US) imaging; the red-to-yellow color map represents microvascular distribution and PA signal amplitude. Red dashed boxes indicate SDMUs in the resting state, while purple dashed boxes indicate SDMUs in the active state. Imaging depth is 1.2 mm, Scale bar: 50 µm.

## 4 Discussions

This study employed a fingerprint-guided dual-modal PA/US microscopy system to systematically investigate the spatial mapping and functional correlation of SDMUs in human fingertips under exercise-induced thermal stress. The findings not only confirm the synergistic activation of sweat ducts and surrounding microvessels during heat stress but also establish the SDMU as a unified spatially coupled functional unit. Furthermore, this coupled unit holds promise as a novel quantitative biomarker for the early detection and assessment of diabetic neuropathy.

### 4.1 Exercise-induced heat stress: overall responses and mechanisms of sweat ducts and microvessels

Consistent with previous studies on cutaneous thermoregulation and sweat duct morphology [24], our results indicate that the segmented US-visible sweat ductal/tubular structures undergo significant post-exercise morphological changes, characterized by increased equivalent diameter, length, and volume (*P* < 0.0001). These changes support the sensitivity of ultrasound-derived ductal/tubular metrics to exercise-induced physiological stress. However, they should not be interpreted as direct evidence of whole-gland enlargement or secretory-coil expansion. More importantly, the vascular network exhibited significantly greater density (*P* < 0.001), while there was no obvious difference in mean microvascular diameter or count. This suggests that exercise-induced fingertip hyperemia is primarily reflected by increased recruitment or representation of perfused microvascular structures rather than uniform dilation of existing blood vessels.

An anatomical distinction should be noted when interpreting the ultrasound-derived sweat structure metrics. The eccrine sweat duct comprises a deeper coiled secretory portion and a ductal component that extends toward the skin surface [24], [25]. The secretory coil is primarily responsible for sweat production, whereas the ductal pathway conducts the secreted fluid toward the skin surface and contributes to final sweat composition through ductal modification and reabsorption [37]. Therefore, the volume, equivalent diameter, and length quantified in Fig. 10 should be interpreted as metrics of US-visible ductal/tubular structures rather than direct measurements of the entire eccrine gland or the secretory coil. The observed post-exercise changes may reflect ductal/tubular deformation, luminal filling, or local tissue-geometry changes accompanying active sweating, but the present ultrasound dataset cannot distinguish these mechanisms or independently quantify secretory-coil expansion. Future work will further optimize the present PA/US platform for depth-resolved visualization of deeper eccrine structures, particularly the secretory coil located in the deep dermis, together with co-registered microvascular assessment, to better determine how exercise-induced US-visible ductal changes relate to whole sweat duct responses and local vascular recruitment during active sweating [25], [40].

### 4.2 SDMU: A unified functional unit with defined spatial boundaries and strong correlative activation

A key innovation of this study lies in establishing the SDMU as an independent functional unit using a dual-mode imaging system and a non-rigid registration framework guided by ultrasound fingerprints. A local photoacoustic vascular template matching algorithm effectively eliminates registration errors caused by finger tremors and post-movement vasodilation, thereby ensuring that quantitative analysis is performed exclusively on the same SDMU under both resting and thermal stress conditions—a prerequisite for investigating its spatial and functional coupling.

Our spatial boundary analysis identified the optimal radial distance for microvascular coupling as approximately 75 μm from the sweat ductal/tubular structure (R5 radius), where microvascular recruitment was most pronounced and consistent. Furthermore, the strongest vascular response was observed at a physical depth of 1100 μm, defining the vertical imaging range used for SDMU analysis. These findings should be interpreted as an optimized imaging window in which US-visible ductal/tubular changes are most strongly associated with PA-detectable microvascular recruitment, rather than as a direct anatomical boundary of the whole eccrine gland or secretory coil. The depth of 1100 μm is compatible with the deeper dermal location of eccrine gland structures reported in anatomical and three-dimensional reconstruction studies [40], but the present PA/US measurements do not independently segment the secretory coil.

Quantitative correlation analysis revealed significant positive correlations between US-visible ductal/tubular morphological parameters (maximum diameter, average diameter, and three-dimensional volume) and the integrated intensity of the corresponding microvascular region, with volume showing the strongest correlation with integrated intensity (*r* = 0.701, *p* = 0.00013). These results suggest that 3D volume is a sensitive US-derived descriptor of ductal/tubular morphological response because it integrates changes in both diameter and length. More importantly, these correlations indicate that ductal/tubular morphological changes and local microvascular recruitment are spatially associated within the optimized SDMU window. This supports the SDMU as a co-registered imaging-defined functional unit, while avoiding the stronger interpretation that the entire secretory coil was directly visualized or that sweat production was quantitatively measured.

### 4.3 Spatial heterogeneity and selective recruitment: a proof-of-concept evaluation

Building upon the established spatial boundaries, we introduced a quantitative dual-threshold evaluation method to systematically assess the functional status of individual SDMUs. As a proof of concept, applying this evaluation framework to longitudinally track 29 SDMUs in a representative subject revealed significant spatial heterogeneity under heat stress conditions. We found that approximately 70% of these tracked SDMUs (20 out of 29) were selectively activated, exhibiting coordinated increases in US-visible ductal/tubular morphology and microvascular perfusion, while the remaining 30% (9 out of 29) remained dormant, with minimal morphological and vascular changes. This localized observation suggests that individual SDMUs do not respond uniformly during exercise-induced heat stress. Instead, the results indicate an “on-demand” recruitment pattern, in which only a subset of imaging-defined SDMUs shows coordinated ductal/tubular and vascular responses. This heterogeneity may be attributed to local differences in ductal/tubular sensitivity, microvascular density, or sympathetic innervation; these findings are consistent with previous observations that sweat duct recruitment and sweating responses vary across skin regions and individuals [41]. From a functional perspective, the selective activation of SDMUs may enable spatially resolved regulation of heat dissipation.

### 4.4 Limitations, clinical implications, and future directions

Despite the robust methodological framework established in this study, several limitations warrant consideration. First, although the overall physiological responses were assessed across the participant cohort, the detailed single-unit heterogeneity evaluation was concentrated on 29 SDMUs from a single representative subject. Accordingly, the observed 70/30 activation ratio should be interpreted as an exploratory single-participant observation rather than as a population-level estimate of SDMU activation. This limitation is particularly notable because previous studies have reported significant inter-individual differences in both the sudomotor response and the cutaneous vascular response [41]. Future studies will require larger and more diverse cohorts of independent participants, repeated measurements, and subject-level statistical models to quantify intra- and inter-individual variability, validate the physiology-informed operational dual-response criterion, and determine the generalizability of SDMU-derived metrics.

From a clinical translation perspective, the proposed PA/US framework should be viewed as complementary to established methods for assessing sweating and skin microstructure. OCT enables non-invasive, high-resolution visualization of sweat ducts and has been used for structural and dynamic assessment of eccrine sweating [5], [10], [24]. However, conventional structural OCT primarily relies on optical scattering or refractive-index contrast, and simultaneous co-registered assessment of sweat duct morphology and hemoglobin-rich dermal microvasculature remains challenging. The ventilated capsule technique is a well-established method for continuously measuring local sweat rate during thermal or exercise stress [6], [7]. Nevertheless, it provides an area-averaged evaporative output and cannot assess morphological changes in individual sweat duct, nor can it determine how these units are spatially coupled to surrounding microvascular regions.

Compared with these approaches, the main advantage of the present PA/US framework is its ability to integrate ultrasound-derived sweat duct morphology with PA-derived microvascular information within the same three-dimensional coordinate system [15], [20], [21]. This co-registered dual-contrast strategy allows the sweat duct-microvasculature unit (SDMU) to be evaluated as a local functional unit rather than as isolated ductal morphology or bulk sweat output alone. This capability may be particularly relevant for neuropathy-related assessment, because diabetic and autonomic neuropathies can involve both sudomotor dysfunction and microvascular impairment [42], [43]. By simultaneously quantifying sweat ductal/tubular morphology and local microvascular recruitment, the PA/US framework may provide a more spatially resolved readout of neurovascular and sudomotor coupling than either OCT-based structural imaging or ventilated-capsule sweat-rate measurement alone. Nevertheless, because the present study was conducted in healthy participants, the clinical diagnostic value of SDMU-derived metrics remains to be validated in dedicated patient cohorts. Clinical translation will require prospective validation in disease-specific cohorts, assessment of test–retest reproducibility and inter-operator robustness, and establishment of clinically interpretable SDMU response.

Primary focal hyperhidrosis is characterized by excessive sweating beyond thermoregulatory needs, commonly affecting the palms, soles, axillae, or craniofacial region, and can substantially impair patients’ quality of life [44]. However, its diagnosis and severity assessment remain challenging because current evaluation relies largely on medical history and regional sweat-output tests. Although these approaches provide useful clinical evidence, sweating episodes can be intermittent and emotionally triggered, while Minor starch–iodine testing mainly provides qualitative or semi-quantitative spatial mapping of sweating areas rather than objective single-duct functional information [45].

The proposed fingerprint-guided dual-modal PA/US microscopy framework may be extended in future studies to hyperhidrosis cohorts. This extension is physiologically plausible because primary hyperhidrosis has been associated with exaggerated sympathetic cholinergic sudomotor activity, and acetylcholine released from sympathetic cholinergic nerves is a major stimulus for eccrine sweat secretion [46]. Moreover, active sweating is accompanied by increased cutaneous blood flow and local microvascular perfusion, which may provide vascular support for secretory activity and heat dissipation [38]. In this context, co-registered PA/US imaging may enable monitoring and quantification of single-SDMU changes and overall SDMU activation patterns within the palmar or fingertip region, thereby helping determine whether excessive sweating is associated with pronounced sweat duct responses, altered local microvascular coupling, or abnormal spatial heterogeneity of SDMU activation. These features may provide potential quantitative indicators for future assessment of hyperhidrosis. Such applications remain exploratory and require validation against established clinical assessments, sweat-output measurements, and Minor starch–iodine testing.

## 5 Conclusions

In conclusion, we have developed a fingerprint-guided dual-modal PA/US microscopy platform capable of precisely mapping and quantifying the spatial-functional dynamics of SDMUs in vivo. Our results demonstrate that exercise-induced thermal stress elicits a synchronized neurovascular response, revealing that cutaneous hyperemia is primarily driven by targeted microvascular recruitment rather than global vasodilation. By quantitatively delineating the SDMU within a highly localized 75 μm radial boundary and an axial depth of 1100 μm, we verified a robust positive correlation between US-visible ductal/tubular morphological expansion and localized vascular activation. Ultimately, this study transitions the SDMU from a conceptual model to a rigorously defined imaging-based functional unit, deepening our fundamental understanding of cutaneous thermoregulation. Furthermore, this quantitative paradigm may provide a non-invasive framework for the future assessment of microvascular and autonomic anomalies, particularly diabetic peripheral neuropathy.

## CRediT authorship contribution statement

**Zhida Chen:** Writing – original draft, Formal analysis, Data curation, Conceptualization. **Yi Liu:** Project administration, Methodology, Investigation. **Dezi Li:** Validation, Resources. **Zihao Yang:** Supervision, Methodology. **Wangbiao Li:** Supervision, Software. **Meifang Li:** Supervision, Formal analysis. **Jing Wang:** Formal analysis. **Zhifang Li:** Data curation, Conceptualization.

## Ethics declaration

Written informed consent to take part in the study and to publish the article has been obtained from all participants or their legal representatives. The privacy rights of participants have been observed.

This study was performed in compliance with relevant laws, regulatory frameworks and guidelines where the research took place. This study was approved by the Fujian Normal University Special Committee on Human Ethics Review. (Approval No. 2026010)

## Declaration of Competing Interest

The authors declare that they have no known competing financial interests or personal relationships that could have appeared to influence the work reported in this paper.

## Data Availability

Data will be made available on request.
