# Supplementary material for: Quantitative 3D mapping of the integrated sweat duct-microvasculature unit via fingerprint-guided photoacoustic/ultrasound microscopy
Source: Photoacoustics. 2026 Nov 2;52:100894. doi: 10.1016/j.pacs.2026.100894 (PMC13756631; doi:10.1016/j.pacs.2026.100894)
Supplement: Supplementary file 1 [file mmc1.docx]

**Supplementary Information**

**Supplementary Information S1. Parameters for Frangi-based PA vascular image processing**

To ensure reproducibility of the photoacoustic (PA) vascular image enhancement workflow, the fixed preprocessing, Frangi multi-scale vesselness filtering, output fusion, and post-processing parameters used in this study are summarized below. The analysis was performed on two-dimensional en-face PA vascular images on a slice-by-slice basis rather than by direct three-dimensional Frangi filtering. Unless otherwise stated, the listed parameters were fixed before quantitative analysis and applied consistently to all images used for vascular enhancement and vessel descriptor extraction.

**Table S1. Fixed parameters used for Frangi-based PA vascular image enhancement and quantitative vessel analysis.**

| **Processing step** | **Parameter** | **Setting used in this study** | **Implementation / source** | **Reproducibility note** |
| --- | --- | --- | --- | --- |
| Input image | Image dimensionality | Two-dimensional image files processed slice by slice | Each image file was read as a 2D image array | No direct 3D Frangi filtering was applied. |
| Input image | Supported file formats | .png, .jpg, .jpeg, .bmp, .tiff, .tif | supported_formats | Batch processing was restricted to these image extensions. |
| Input image | Color handling | RGB images converted to grayscale using cv2.COLOR_RGB2GRAY; grayscale images used directly | EnhancedVesselDetector.__init__() | All subsequent processing was performed on grayscale images. |
| Spatial calibration | Pixel size | 0.015 mm/pixel (15 um/pixel) | PIXEL_SIZE_MM = 0.015 | Sigma values were specified in pixels and converted to physical scale using this calibration. |
| Preprocessing before Frangi | CLAHE | clip_limit = 0.012; kernel_size = None; nbins = 256 | exposure.equalize_adapthist(gray, clip_limit=0.012); other values followed scikit-image 0.25.2 defaults | The CLAHE-enhanced image was used as the first preprocessing output. |
| Preprocessing before Frangi | Gamma correction | gamma = 1.2; gain = 1 | exposure.adjust_gamma(equalized, gamma=1.2); gain followed the scikit-image 0.25.2 default | Applied after CLAHE and before intensity normalization. |
| Preprocessing before Frangi | Intensity normalization | Min-max normalization to 0-255; dtype = uint8 | ((image - min) / (max - min) * 255).astype(np.uint8) | Standardized the Frangi input image. |
| Frangi vessel enhancement | Function | skimage.filters.frangi in scikit-image 0.25.2 | from skimage.filters import frangi | The software version is reported to support reproducibility. |
| Frangi vessel enhancement | Vessel polarity | black_ridges = False | frangi(..., black_ridges=False) | Used to enhance bright vascular structures on a dark background. |
| Frangi vessel enhancement | Fine-vessel sigma range | range(1, 6, 1) = [1, 2, 3, 4, 5] pixels | range(1, 6, 1) | Equivalent to approximately 15-75 um using the 15 um/pixel calibration. |
| Frangi vessel enhancement | Medium-vessel sigma range | range(3, 11, 2) = [3, 5, 7, 9] pixels | range(3, 11, 2) | Equivalent to approximately 45-135 um. |
| Frangi vessel enhancement | Large-vessel sigma range | range(5, 16, 2) = [5, 7, 9, 11, 13, 15] pixels | range(5, 16, 2) | Equivalent to approximately 75-225 um. |
| Frangi vessel enhancement | Shape/noise parameters | alpha = 0.5; beta = 0.5; gamma = None | scikit-image 0.25.2 frangi parameters used in the analysis environment | These values define the vesselness response and background/noise suppression. |
| Frangi vessel enhancement | Boundary handling | mode = 'reflect'; cval = 0 | scikit-image 0.25.2 default boundary handling | Defines image-border extension and constant fill value. |
| Frangi output fusion | Multi-scale fusion | Pixel-wise maximum projection of the three Frangi response maps | np.maximum.reduce(results) | Fine, medium, and large vesselness response maps were fused after separate Frangi filtering. |
| Frangi output normalization | Vesselness map scaling | Min-max normalization to 0-255; dtype = uint8 | ((vessels_combined - min) / (max - min) * 255).astype(np.uint8) | The normalized vesselness map was used for subsequent segmentation and descriptor extraction. |
| Segmentation after Frangi | Binary vessel mask | Automatic Otsu thresholding of the normalized vesselness map | Otsu thresholding, as described in Fig. 6 workflow | The same thresholding strategy was applied to obtain binary vascular masks for quantitative analysis. |
| Skeleton and diameter analysis | Skeletonization | Single-pixel vascular centerlines extracted from the binary mask | Vascular skeletonization workflow | Skeletons were used for topological descriptors and vessel-centerline representation. |
| Skeleton and diameter analysis | Diameter estimation | Exact distance transform applied to the binary vessel mask and sampled along the skeleton | Distance transform-based diameter heatmap workflow | Diameter maps were used to quantify local vessel diameter distributions. |
| Software environment | Version information | Python 3.12.4; NumPy 2.2.6; OpenCV/cv2 4.12.0; Pillow 11.2.1; Matplotlib 3.10.3; scikit-image 0.25.2 | Local package-version check performed on 2026-08-06 | Versions are reported to facilitate independent reproduction of the workflow. |

*Note: Parameters related to thresholding, skeletonization, and diameter estimation are included because these steps were performed after Frangi enhancement to generate the quantitative vessel descriptors reported in the manuscript.*

**Fig.S1. Quantitative evaluation of U-Net training over 200 epochs**. **a,** Loss convergence analysis. Solid lines represent the original training (red) and validation (orange) losses, while dashed lines indicate the smoothed trends. The rapid decline and subsequent stabilization of the validation loss confirm the model's generalization ability, with no obvious overfitting observed. **b,** Evolution of segmentation accuracy. This curve shows the mean Intersection over Union (MioU) on the training set, which rises sharply and plateaus at approximately 98.5%, demonstrating the model's high fidelity in capturing fingertip boundaries and sweat duct features.
